# Analyses of an epigenetic switch involved in the activation of pioneer factor FOXA1 leading to the prognostic value of estrogen receptor and FOXA1 co-expression in breast cancer

**DOI:** 10.18632/aging.102250

**Published:** 2019-09-28

**Authors:** Xuan Jing, Hongping Liang, Chonghua Hao, Li Hongxia, Xiangrong Cui

**Affiliations:** 1 Department of Clinical Laboratory, Shanxi Provincial People's Hospital, Affiliate of Shanxi Medical University, Taiyuan 030001, P.R. China; 2 Reproductive Medicine Center, Children's Hospital of Shanxi and Women Health Center of Shanxi, Affiliate of Shanxi Medical University, Taiyuan, Shanxi 030000, P.R. China; 3 Department of Oncology, Shanxi Provincial People's Hospital, Affiliate of Shanxi Medical University, Taiyuan 030001, P.R. China

**Keywords:** breast cancer, FOXA1, methylation, hormone receptor, prognosis, aging, age-related diseases

## Abstract

Forkhead box protein A1 (*FOXA1*) is a pioneer factor of estrogen receptor α (ER)–chromatin binding and function, yet the role of *FOXA1* in breast cancer and the underlying molecular mechanisms have not yet been elucidated. To evaluate gene expression alterations during breast carcinogenesis, *FOXA1* expression was analyzed using the Serial Analysis of Gene Expression Genie suite, a gene expression profiling interactive analysis, and Oncomine analyses. The correlation between methylation and expression was analyzed using the MEXPRESS tool and UCSC Xena browser. Then, the expression and prognostic value of FOXA1 was validated by our own breast cancer samples using RT-PCR. We obtained the following important results. (1) The expression level of *FOXA1* was significantly higher in breast cancer than normal tissues. (2) ER, PR, HEGR-2, and nodal status were positively correlated with *FOXA1* expression. (3) Among patients with ER+ tumors, those with higher *FOXA1* expression levels had better survival probabilities. (4) The major mutation type in *FOXA1* in breast cancer samples was missense mutations. (5) *FOXA1* expression was significantly higher in ER+ breast tumors than in ER− tumors or normal tissues. Our findings suggest that the aberrant DNA hypomethylation of promoter regions is one mechanism underlying the aberrant expression of *FOXA1* in ER+ breast cancer, which might be a potential indicator of favorable prognosis.

## INTRODUCTION

Breast cancer is the most frequent cancer in women worldwide and the fifth leading cancer-related cause of death, with about 1.7 million cases diagnosed and about 0.5 million associated deaths globally in 2011 according to the World Health Organization [1]. Several clinicopathological features, such as tumor size, histological subtype and grade, lymph node metastases, hormone receptor status, and the expression of human epidermal growth factor receptor-2 (HER-2), have been implemented in breast cancer management [2]. While these parameters reflect the biological features of the cancer and the patient, they are not adequate for predicting the prognosis of individual patients [3–5]. Therefore, the identification of specific and sensitive prognostic factors and therapeutic targets in breast cancer has clinical significance.

The expression of ER is a crucial prognostic and predictive factor in breast cancer and is important in biological research related to breast cancer. Previous studies have reported that in estrogen receptor α (ER)-positive (ER+) cells, androgens inhibit cell proliferation [6, 7], whereas in ER-negative (ER−) breast cancer cells, androgens activate cell proliferation [8]. However, not all ER+ breast cancers behave in the same way [9]. Understanding why and how ER+ breast cancers differ is important from research and clinical perspectives.

Forkhead box protein A1 (FOXA1; also known as hepatocyte nuclear factor 3α or HNF3A), was originally identified as a transcriptional activator in liver development and is expressed in breast cancer [10, 11]. FOXA1 binds to target sites in silent chromatin and triggers transcriptional competency by initial chromatin de-compaction [11]. It can directly bind to the estrogen receptor 1 promoter and modulate ER activity [12]. In addition, it has prognostic value for breast cancer; FOXA1 expression in ER+ breast cancer is positively correlated with better prognosis [13]. However, the precise role of FOXA1 in breast cancer and the molecular mechanisms underlying its effects have not been elucidated.

In this report, we hypothesized that FOXA1 is a promising candidate as a therapeutic and prognostic target for breast cancer. To evaluate this hypothesis, we used a bioinformatics approach to determine the expression and prognostic value of *FOXA1* in breast cancer overall and in its subtypes. Furthermore, we identified the mutation and methylation status of *FOXA1* in breast cancer to improve the characterization of malignant cells and thereby predict treatment response and prognosis. Our results demonstrated that the expression of *FOXA1* is affected by methylation and ER+ tumor status and is related to prognosis in breast cancer. These findings will contribute to the development of novel therapeutics for breast cancer.

## RESULTS

### *FOXA1* transcript expression status in human breast cancer

**Figure 1:**
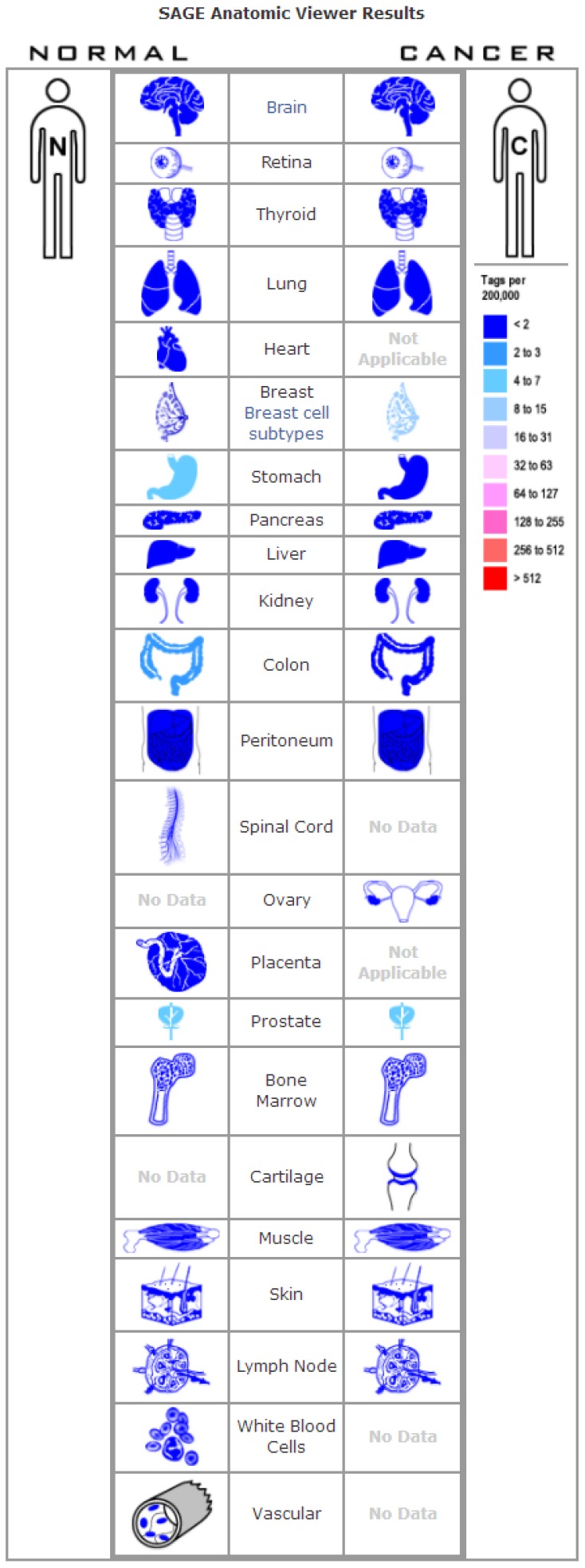
**Digital FOXA1 gene expression profiles were analyzed and displayed using the SAGE Anatomic Viewer.**

**Figure 2:**
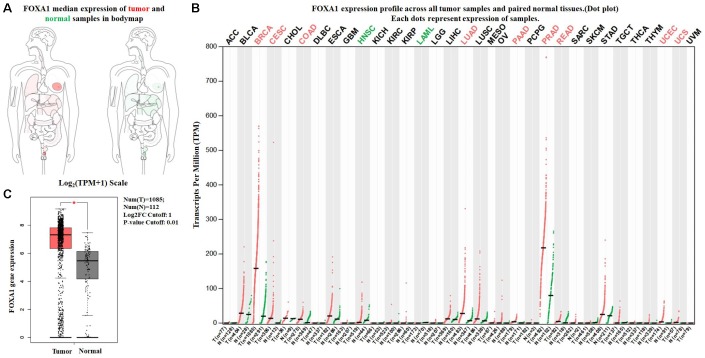
**Expression of *FOXA1* in breast cancer and normal tissues from GEPIA.** (**A**) *FOXA1* median expression of tumor (red) and normal (green) samples in bodymap. (**B**) *FOXA1* epxression profile across all tumor (red) and paired normal (green) tissues. Each dot represents the expression of sample. (**C**) The expression of *FOXA1* mRNA in breast cancer tissues (red box) and paired normal tissues (black box) from GEPIA.

**Figure 3:**
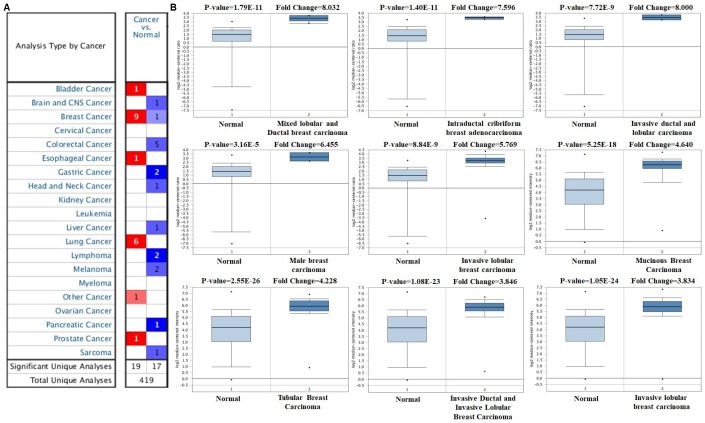
***FOXA1* analysis in breast cancer (Oncomine database).** The online Oncomine analysis tool (red: overexpression, blue: down-expression) was used to compare *FOXA1* expression levels in breast cancer specimens with matched normal specimens. The thresholds for significant probes for each microarray dataset included a two-fold difference in expression between cancer and normal samples and *P* < 0.0001. The box plot compares *FOXA1* expression in cancer samples (right) and matches normal (left) samples generated from the Oncomine database.

The expression profile of *FOXA1* was identified using the SAGE Digital Gene Expression Display. SAGE data showed that *FOXA1* was overexpressed in breast cancer tissues compared with matched normal tissues (Figure 1). Using GEPIA, we found that the expression level of *FOXA1* was significantly higher in breast cancer, cervical squamous cell carcinoma, endocervical adenocarcinoma, colon adenocarcinoma, lung adenocarcinoma, pancreatic adenocarcinoma, prostate adenocarcinoma, rectum adenocarcinoma, uterine corpus endometrial carcinoma, and uterine carcinosarcoma than in their matched normal tissues (Figure 2). To further confirm this result, the Oncomine database was used to assess the expression profile of *FOXA1*. Elevated mRNA levels of *FOXA1* were identified in various human tumors, including bladder cancer, breast cancer, esophageal cancer, lung cancer, and prostate cancer (Figure 3A). *FOXA1* expression was significantly higher in mixed lobular and ductal breast carcinoma, intraductal cribriform breast adenocarcinoma, invasive ductal and lobular carcinoma, male breast carcinoma, invasive lobular breast carcinoma, mucinous breast carcinoma, tubular breast carcinoma, invasive ductal, and invasive lobular breast carcinoma than in normal samples (Table 1, Figure 3B).

**Table 1:** Foxa1 expression in breast cancer.

**Cancer subtype**	***p*-value**	**Fold change**	***t*-test**	**Rank (%)**	**Sample**	**Reference**
Mixed lobular and ductal breast carcinoma	1.79E-11	8.032	7.938	1	7	TCGA
Intraductal cribriform breast adenocarcinoma	1.40E-11	7.596	8.108	1	3	TCGA
Invasive ductal and lobular carcinoma	7.72E-9	8.000	7.460	2	3	TCGA
Male breast carcinoma	3.16E-5	6.455	5.875	4	3	TCGA
Invasive lobular breast carcinoma	8.84E-9	5.769	6.184	8	36	TCGA
Mucinous breast carcinoma	5.25E-18	4.640	10.155	2	46	[24]
Tubular breast carcinoma	2.55E-26	4.228	12.141	2	67	[24]
Invasive ductal and invasive lobular breast carcinoma	1.08E-23	3.846	11.159	4	90	[24]
Invasive lobular breast carcinoma	1.05E-24	3.834	11.361	7	148	[24]

### *FOXA1* mutations in breast cancer

**Figure 4:**
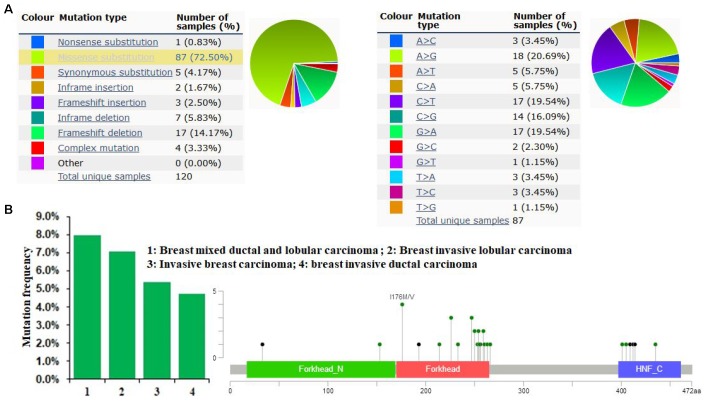
***FOXA1* mutations in human breast cancer.** (**A**) The pie chart generated using COSMIC summarizes the observed mutation types, including nonsense substitutions, missense substitutions, synonymous substitutions, in-frame insertions, frameshift insertions, in-frame deletions, frameshift deletions, and complex mutations. (**B**) As determined using cBioPortal, the *FOXA1* mutation frequency was less than 8% in patients with breast cancer. A total of 33 mutation sites were detected between amino acids 0 and 472. The *FOXA1* mutation occurs primarily in the Forkhead domain.

The pie chart in Figure 4 generated using COSMIC summarizes the observed mutation types, including nonsense substitutions, missense substitutions, synonymous substitutions, in-frame insertions, frameshift insertions, in-frame deletions, frameshift deletions, and complex mutations. Mutations in breast cancer samples included 0.83% nonsense substitutions, 72.5% missense substitutions, 4.17% synonymous substitutions, 1.67% in-frame insertions, 2.5% frameshift insertions, 5.83% in-frame deletions, 14.17% frameshift deletions, and 3.33% complex mutations (Figure 4A). *FOXA1* mutations in breast cancer samples were 20.69% A > G, 19.54% C > T, 19.54% G > A, and 16.09% G > C (Figure 4A). As determined using cBioPortal, the *FOXA1* mutation frequency was less than 8% in patients with breast cancer. A total of 33 mutation sites were detected between amino acids 0 and 472. FOXA1 mutations mainly occurred in the Forkhead domain (Figure 4B).

### Genetic alterations in *FOXA1* and clinicopathological parameters

**Figure 5:**
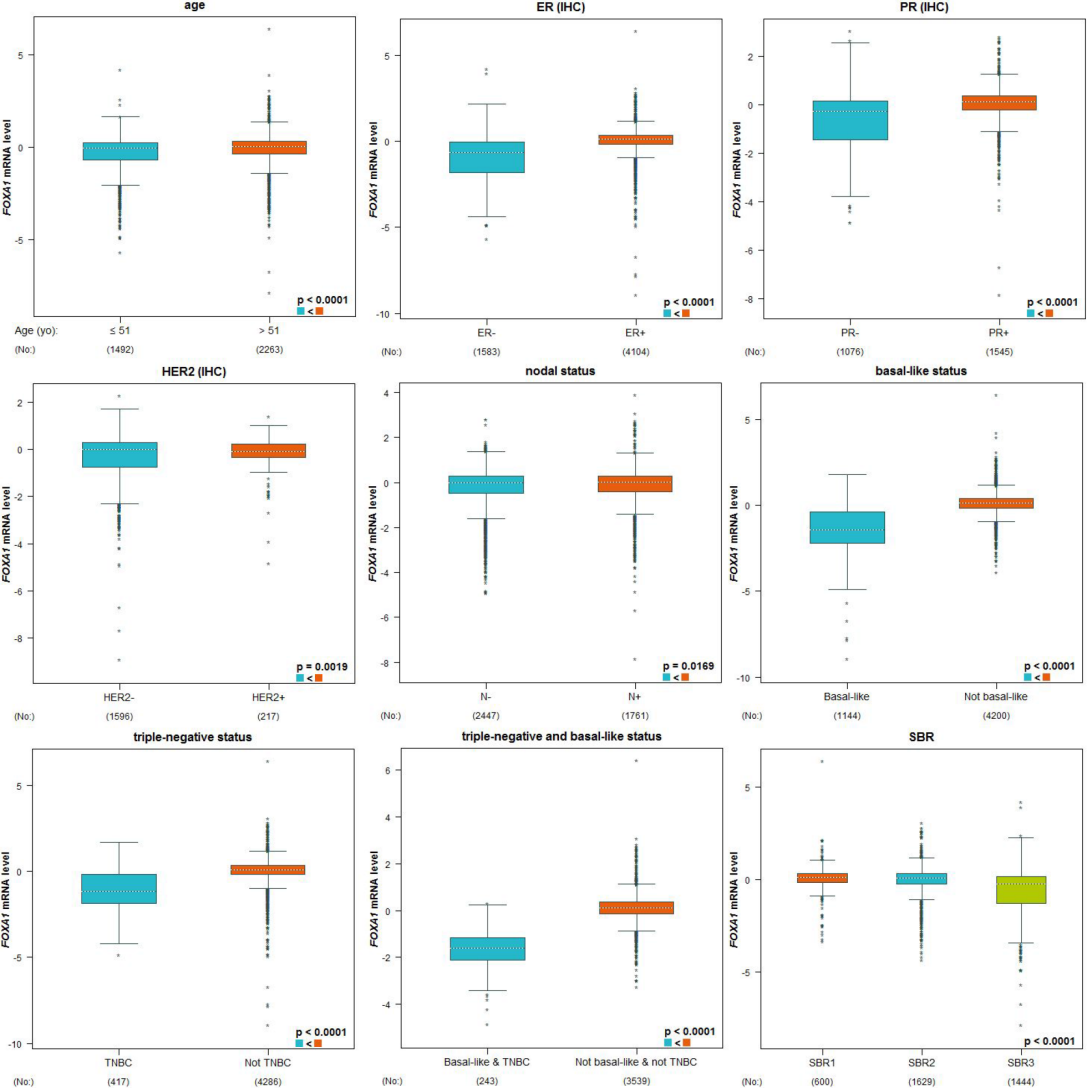
**Genetic alterations in *FOXA1* and clinicopathological parameters.** Based on clinical pathology parameters, the expression profile of *FOXA1* was expressed in the PAM50 breast cancer subtype using 5861 patients in bc-GenExMiner 4.0. A globally significant difference between the groups was assessed by Welch's t-test to generate *p*-values, as well as the Dunnett-Tukey-Kramer test.

**Figure 6:**
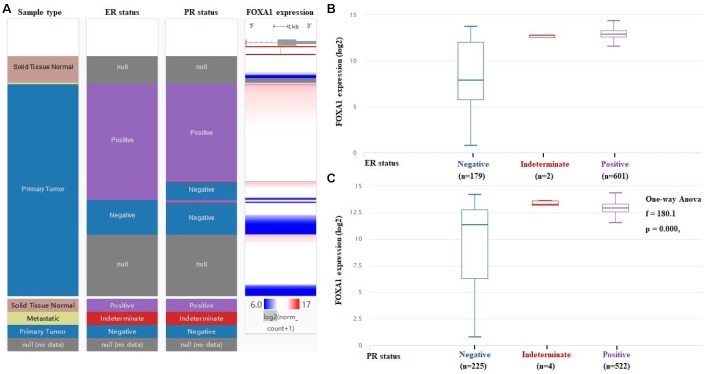
***FOXA1* expression varies significantly among ER and PR status of breast cancer.** (**A**) The heatmap of *FOXA1* expression in ER and PR status of breast cancer. (**B**) The box blots of *FOXA1* expression in ER status of breast cancer. (**C**) The box blots of *FOXA1* expression in PR status of breast cancer.

**Figure 7:**
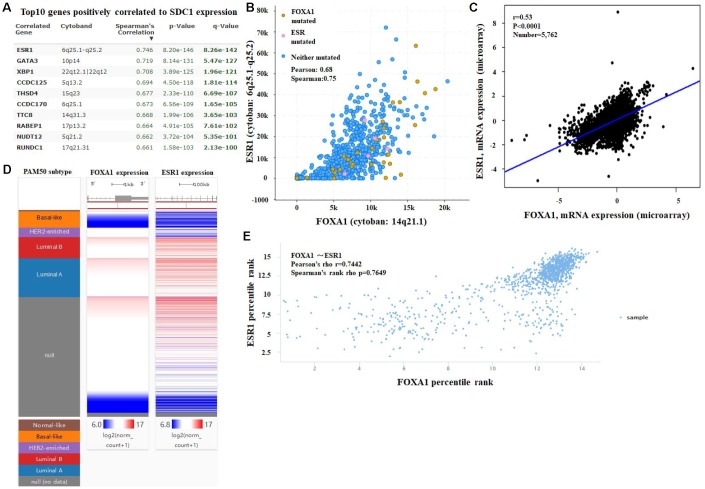
(**A**) Co-expression of the *FOXA1* gene as determined by cBioPortal. (**B**) Regression analysis between *FOXA1* and *ESR1* in breast cancer performed by cBioPortal. (**C**) Relationship between *FOXA1* and *ESR1* in breast cancer determined through bc-GenExMiner v4.0. (**D**) Heat map of *FOXA1* and *ESR1* mRNA expression across PAM50 breast cancer subtypes in TCGA database, identified by UCSC Xena. (**E**) Correlation between *FOXA1* and *ESR1* mRNA expression in the TCGA database, identified by UCSC Xena.

We examined the expression profile of *FOXA1* across PAM50 breast cancer subtypes using 5861 patients in bc-GenExMiner 4.0 based on clinical-pathological parameters. Regarding age, *FOXA1* mRNA levels were remarkably higher in patients >51 years old than in patients ≤51 years old (Table 2, Figure 5). ER, progesterone receptor (PR) status, HER-2, and nodal status were positively correlated with *FOXA1* expression (Table 2, Figure 5). Triple-negative breast cancer (TNBC) is negative for ER, PR, and HER-2. *FOXA1* mRNA expression was significantly downregulated in patients with TNBC (P < 0.0001) compared with that in the group without TNBC (Table 2 and Figure 5). Furthermore, patients with negative basal-like characteristics exhibited significantly higher *FOXA1* expression than that in patients with basal-like characteristics (P < 0.0001) (Table 2, Figure 5). A more advanced Scarff Bloom and Richardson grade status (SBR) grade was associated with lower *FOXA1* mRNA levels (Figure 5). We examined the expression profile of *FOXA1* in different breast cancer (BRCA) subtypes in TCGA-BRCA using the UCSC Xena browser. A heatmap and corresponding box plots revealed that the ER+ and PR+ subtypes had higher *FOXA1* mRNA expression levels than those of their corresponding negative subtypes (Figure 6). To further investigate the regulatory mechanisms underlying the role of FOXA1 in breast cancer, data mining was conducted for a breast cancer cohort using cBioPortal. *ESR1* is a highly correlated gene (Figure 7A); it drives growth in the majority of human breast cancers by binding to regulatory elements and inducing transcriptional events that promote tumor growth [14]. A regression analysis revealed that *FOXA1* and *ESR1* levels are highly correlated (Pearson’s correlation = 0.68; Spearman’s correlation = 0.75) (Figure 7B). The positive correlation between *FOXA1* and *ESR1* mRNA expression was identified using data from the bc-GenExMiner 4.0 database (Figure 7C). By investigating breast cancer data in TCGA using UCSC Xena, the positive correlation was confirmed (Figure 7D and 7E). These data demonstrated that *FOXA1* could be related to the ESR1 pathway in breast cancer.

**Table 2:** The relationship between mRNA expression of FOXA1 and clinicopathological parameters of breast carcinoma.

**Variables**	No.*	**mRNA**	***p*-value**
**Age**			
≤ 51	1492	-	< 0.0001
> 51	2263	↑	
**Nodal status**			
-	2447	-	0.0169
+	1761	↑	
**ER (IHC)**			
-	1583	-	< 0.0001
+	4104	↑	
**PR (IHC)**			
-	1076	-	< 0.0001
+	1545	↑	
**HER2 (IHC)**			
-	1596	-	0.0019
+	217	↑	
**Triple-negative Status**			
Not	4286	-	< 0.0001
TNBC	417	↓	
**Basal-like Status**			
Not	4200	-	< 0.0001
Base-like	1144	↓	

### FOXA1 expression is upregulated by hypomethylation in ER+ breast tumors, as determined by in silico analyses

**Figure 8:**
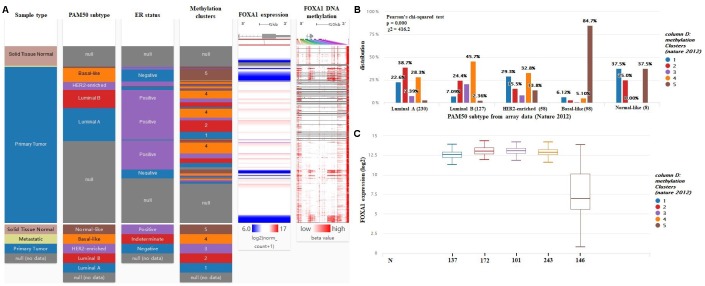
***FOXA1* expression is negatively regulated by DNA methylation.** (**A**) Heatmap including BRCA PAM50 subtypes, ER status, and BRCA DNA methylation data from TCGA-BRCA were identified by the UCSC Xena browser. (**B**) DNA methylation patterns in different subtypes of breast cancer (cluster 1 to 5, the lowest to the highest). (**C**) The expression of *FOXA1* in different BRCA DNA methylation clusters.

**Figure 9:**
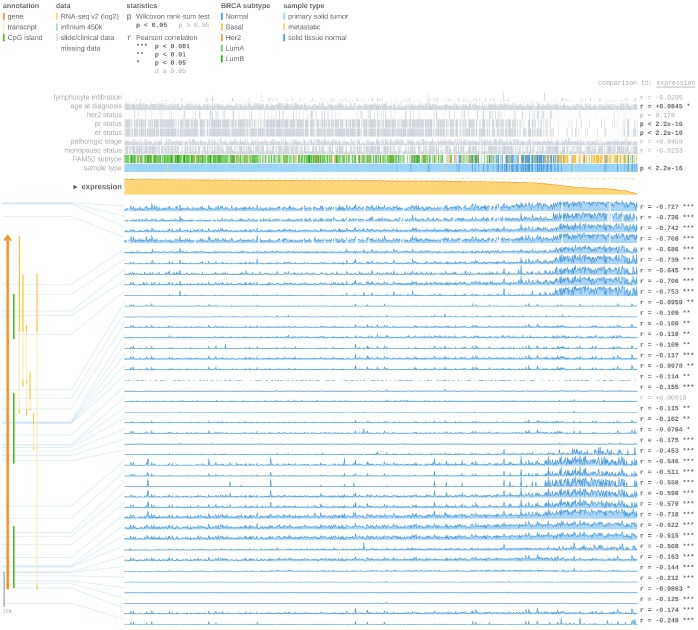
***FOXA1* expression and methylation status in breast cancer using MEXPRESS tool.** At the top of the figure, clinical TGCA data is displayed and classified according to *FOXA1* expression. On the right side, the Pearson’s correlation coefficient r and p values for Wilcoxon rank-sum test are displayed. The *FOXA1* expression is represented by the orange line in the center of the graph. According to the expression of *FOXA1*, the highest expression was found on the left side and the lowest on the right side. The blue lines (lower right) represent the Infinium 450 k probes linked to *FOXA1*. *FOXA1* gene and CpG islands (green lines) are displayed on the left side (bottom).

Using the UCSC Xena browser, we generated a heatmap that included BRCA PAM50 subtypes, ER status, and BRCA DNA methylation data from TCGA-BRCA (Figure 8A). We found that the basal-like subtype has the highest level of DNA methylation, while the luminal A subtype has the lowest level of DNA methylation (Figure 8A). In addition, most ER+ cases were enriched in the luminal A subgroups (55.5%) (Figure 8A). The basal-like subtype had the highest frequency of the cluster 5 (84.7%) DNA methylation pattern, while the luminal A subtype was most highly represented by cluster 2 (38.7%) and cluster 1 (22.6%) DNA methylation patterns (Figure 8B). Then, we compared the expression of *FOXA1* in different DNA methylation clusters, and we confirmed that *FOXA1* expression decreased gradually as DNA methylation increased (Figure 8C). Therefore, we hypothesized that *FOXA1* expression is regulated by DNA methylation. To verify this hypothesis, we used the MEXPRESS tool. As shown in Figure 9, we detected the methylation of FOXA1 using 40 probes distributed in different regions of the gene (the localization of each probe is presented in the figure, and those localized in the promoter region are highlighted in dark blue). We found that the methylation values increase as tumors become ER−. All regions analyzed revealed a negative correlation with respect to *FOXA1* gene expression (Pearson’s correlation coefficients for each probe are indicated on the right in Figure 9, suggesting that *FOXA1* methylation silences gene expression. Another interesting observation is that the ER status and *FOXA1* expression exhibited a positive correlation; *FOXA1* expression gradually diminished (due to promoter methylation) as tumors became ER− (P < 0.0001). The MEXPRESS tool also allowed us to visualize *FOXA1* expression and the methylation status according to the PAM50 breast cancer molecular classification. As shown in Figure 9, the methylation of FOXA1 was decreased (and FOXA1 expression increases) in luminal A and luminal B subtypes (both ER−, represented as green lines in Figure 9) and increased (with decreased expression) in basal-like and normal-like subtypes (both ER+, represented in Figure 9 as yellow and blue lines, respectively). Since the aberrant DNA hypomethylation of promoter regions is one of the mechanisms underlying the aberrant expression of oncogenes in tumors, and the *FOXA1* promoter is mostly methylated in ER− tumors, we speculate that *FOXA1* functions principally as an oncogene in ER+ breast cancer.

### Relationship of *FOXA1* expression and prognosis in breast cancers

**Figure 10:**
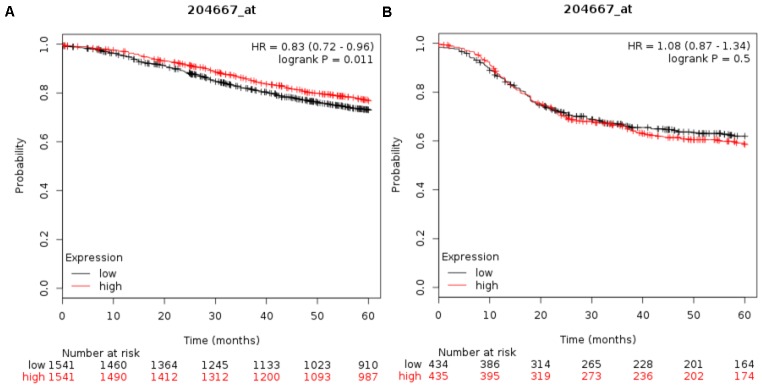
**Relationship of *FOXA1* expression and prognosis in breast cancers.** Recurrence-free survival (RFS) curves calculated by Kaplan-Meier plotter for tumor patients with ER + (**A**) and ER- (**B**), respectively. Survival probability is displayed on the y-axis, time (in months) on the x-axis. Black curves represent low *FOXA1* expression, and red curves represent high *FOXA1* expression. It can be noted that enhanced expression of FOXA1 leads to differences in RFS only in the ER + background (Panel **A**).

**Figure 11:**
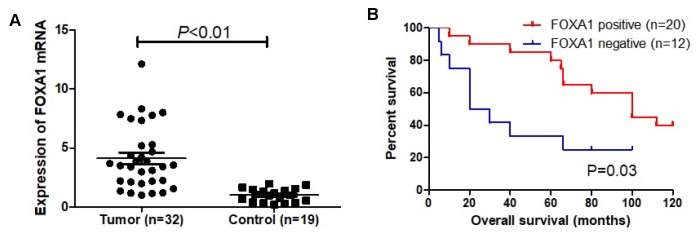
***FOXA1* as a prognosis marker in breast cancer.** (**A**) Expression of FOXA1 in tumor (32 cases) and adjacent normal mammary epithelium (19 cases). (**B**) Kaplan-Meier curves based on FOXA1 expression were drawn for overall survival in 32 patients.

To evaluate whether the expression level of *FOXA1* has predictive value for breast cancer prognosis, we used the online survival analysis software Kaplan–Meier plotter. This tool allowed us to analyze the expression of FOXA1 as dichotomized values (i.e., high expression and low expression based on the median expression). The relationship between *FOXA1* expression and recurrence-free survival for 3779 patients with breast cancer was analyzed, separating ER+ (n = 2565) from ER− (n = 1214) cases. As shown in Figure 10A, among the patients with ER+ tumors, those with higher *FOXA1* expression levels presented better probabilities of survival (P = 0.011). This was not observed for ER− tumors (Figure 10B) (P = 0.49). Our own results demonstrated the upregulation of FOXA1 mRNA expression and better probabilities of survival in breast cancer (Figure 11).

## DISCUSSION

FOXA1, a pioneer transcription factor, binds to heterochromatic DNA on the nucleosome core, forming an open chromatin configuration that facilitates the binding of other transcription factors to gene promoters [15–17]. FOXA1 is the primary determinant of ER binding and transcriptional activity in breast cancer cells and therefore is probably related to the response to endocrine therapy [12, 18]. Furthermore, FOXA1 may have a repressive effect on breast cancer growth by regulating the expression of E-cadherin and cell cycle-dependent kinase inhibitor p27 [19, 20]. These findings suggest that high FOXA1 expression in patients with breast cancer may be correlated with better prognosis.

To determine the role of FOXA1 in the development, progression, and prognosis of breast cancer, we analyzed extensive gene expression data with well-defined parameters in breast cancer and normal samples. Using SAGE and GEPIA, we found that the expression level of *FOXA1* is significantly higher in breast cancer tissues than in normal breast tissues. Using Oncomine, we determined that FOXA1 is overexpressed in mixed lobular and ductal breast carcinoma, intraductal cribriform breast adenocarcinoma, invasive ductal and lobular carcinoma, male breast carcinoma, invasive lobular breast carcinoma, mucinous breast carcinoma, tubular breast carcinoma, invasive ductal, and invasive lobular breast carcinoma. According to the expression status of the ER, PR and HER2, breast cancers are classified as luminal A (ER+ or PR+/HER2-), luminal B (ER+ or PR+/HER2+), HER2 (ER-/PR-/HER2+) or triple-negative breast cancer (TNBC, ER-/PR-/HER2-). TNBC is further subclassified as basal-like breast cancer (BLBC, ER-/PR-/HER2-/CK5/6+ or epidermal growth factor receptor [EGFR] +) and quintuple-negative breast cancer (ER-/PR-/HER2-/CK5/6-/EGFR-) and accounts for 15–25% of all breast cancer cases. Using the bc-GenExMiner 4.0 online database, we demonstrated that ER, PR, HER-2, and nodal status are positively correlated with *FOXA1* expression. Conversely, basal-like status, TNBC status, and SBR were negatively correlated with *FOXA1*. Due to BLBC is a subclass of TNBC, there were great discrepancies in the expression of FOXA1 in these two types of breast cancer. Data from the UCSC Xena browser further confirmed that the ER+ and PR+ subtypes have higher FOXA1 mRNA expression levels than those of their matched negative subtypes. We further demonstrated that FOXA1 could be related to the ESR1 pathway in breast cancer. A survival analysis revealed that higher FOXA1 expression levels were related to better probabilities of survival for patients with ER+ tumors but not those with ER− tumors.

Somatically acquired inherited, epigenetic, transcriptomic, and proteomic alterations are the major four major modifications, as summarized previously [21]. Somatic loss-of-function or gain-of-function alterations occur in specific genomic regions, which could indicate their potential inhibitory or carcinogenic roles [22, 23]. Therefore, the frequencies of alterations and mutations in *FOXA1* were analyzed using the COSMIC and cBioPortal databases. The major mutation type in *FOXA1* was missense mutations. However, a low *FOXA1* alteration frequency was observed in breast cancer. We investigated the mechanisms underlying FOXA1 dysregulation. By examining its DNA methylation status in TCGA-BRCA, we observed a negative correlation between the methylation status of some CpG sites and *FOXA1* expression. The luminal A subtype had the lowest level of overall DNA methylation and the highest *FOXA1* expression. In contrast, the basal-like subtype had the highest level of overall DNA methylation and the lowest *FOXA1* expression. In addition, we confirmed that most ER+ cases were enriched in the luminal A subgroups. A MEXPRESS analyses further indicated that FOXA1 expression is significantly upregulated in ER+ breast tumors compared with ER− tumors or normal tissues. We show that aberrant DNA hypomethylation of promoter regions is one of the mechanisms underlying the aberrant expression of FOXA1 in ER+ breast cancer.

In this study, we identified the significance of FOXA1 expression in human ER+ breast cancer and demonstrated the role of DNA methylation. High *FOXA1* expression and low *FOXA1* DNA methylation in the ER+ subtype of breast cancer are potential indicators of favorable prognosis. This study was hypothesis-driven and performed using experimentally generated data available in public databases, emphasizing the need for future experimental verification of the *FOXA1* regulatory mechanism.

## CONCLUSIONS

We used online databases to successfully integrate various *FOXA1*-related microarray datasets. Based on our results, we conclude that the expression level of *FOXA1* is significantly higher in breast cancer than in noncancerous tissues, and the effects of *FOXA1* could be mediated by the ESR1 pathway. Furthermore, we demonstrated that among patients with ER+ tumors, those with higher *FOXA1* expression levels had better probabilities of survival. Finally, our results suggest that aberrant DNA hypomethylation of promoter regions contributes to the aberrant expression of *FOXA1* in ER+ breast cancer and may be an indicator of favorable prognosis.

## METHODS

### Patients

This study enrolled a consecutive series of 52 patients with primary invasive breast cancer from the middle area of China. As a control, we used 10 non-tumoral-adjacent tissues. Frozen tissues were collected at the Department of Oncology from Shanxi Provincial People's Hospital (China). The mean age is 52.28 years, with an age range of 31 to 82 years.

### Reverse transcription-quantitative polymerase chain reaction (RT-qPCR) analysis

Total RNA was extracted using TRIzol reagent, and cDNA synthesis was conducted with a PrimeScript^TM^ RT Master Mix kit, according to the manufacturer’s protocols (Takara Biotechnology Co., Ltd., Dalian, China). RT-qPCR was performed using SYBER^®^ Premix Ex Taq Kit (Takara Bio, Inc., Tokyo, Japan), according to the manufacturer’s protocol using gene-specific primers, and products were measured on a CFX96 Real-time PCR system (Bio-Rad Laboratories, Inc., Hercules, CA, USA). The primers used were as follows: FOXA1, forward, 5′-CGCTTCGCA CAGGGCTGGAT-3′, and reverse, 5′-TG CTGACCGGGACGGAGGAG-3′. GAPDH (forward:5′- GCTCTCTGCTCCTCCTGTTC-3′ and reverse:5′-CGC CCAATACGACCAAATCC-3′) was used as the endogenous housekeeping gene for normalization of mRNA levels. The results are expressed as the mean of 2^−∆∆Cq^ ± standard deviation. The PCR amplification was performed as follows: 95°C for 5 min; 35 cycles of 95°C for 1 min, 60°C for 1 min and 72°C for 1 min; and then 72°C for 7 min.

### Serial analysis of gene expression

Published serial analysis of gene expression (SAGE) data was performed to analyze *FOXA1* gene expression in normal and malignant human tissues. Digital *FOXA1* gene expression profiles were analyzed and displayed using the SAGE Anatomic Viewer (https://www.ncbi.nlm.nih.gov/SAGE/).

### GEPIA

GEPIA (Gene Expression Profiling Interactive Analysis; http://gepia.cancer-pku.cn/), a web-based tool with fast and customizable features based on The Cancer Genome Atlas (TCGA) data for analysis of the key interactive gene expression profiles for *FOXA1* [25].

### Oncomine database analysis

The Oncomine database (https://www.oncomine.org) is a publicly accessible online data mining platform for collecting, standardizing, analyzing and providing cancer microarray information for biomedical research [26]. The online Oncomine analysis tool was used to compare *FOXA1* expression levels in breast cancer specimens with matched normal specimens. The thresholds for significant probes for each microarray dataset included a two-fold difference in expression between cancer and normal specimens and *P* < 0.0001. The co-expression profile of *FOXA1* in breast cancer was assessed and a heatmap was generated to visualize the results.

### cBioPortal database analysis

The cBio Cancer Genomics Portal (cBioPortal) is a publicly accessible resource (http://www.cbioportal.org/) [27, 28], which provides visualization and analysis tools for more than 5,000 tumor samples from 232 cancer studies in the TCGA pipeline. The term “FOXA1” was used to search the cBioPortal database and The Breast Invasive Carcinoma (TCGA, Cell 2015, n = 818) cohort was utilized. The search parameters included mutations and putative copy-number alterations from GISTIC.

### COSMIC analysis for *FOXA1* mutations

The COSMIC (Catalogue of Somatic Mutations in Cancer) database (https://cancer.sanger.ac.uk), a high-resolution resource for seeking targets and trends in the genetics of human cancer, was used to identify mutations in *FOXA1*. An overview of the distribution of mutations and substitution types on the codogenic strand in breast cancer specimens was generated, and the results are presented in a pie chart [29].

### Breast cancer gene-expression miner v4.1

bcGenExMiner v4.1 (Breast Cancer Gene-Expression Miner v4.1), an online platform for gene expression, prognostic, and correlation analyses in breast cancer, was performed to assess *FOXA1* expression in breast cancer [30, 31]. Correlations between *FOXA1* and the estrogen receptor alpha gene (*ESR1*) were assessed using the bc-GenExMiner v4.1 correlation module.

### University of California Santa Cruz cancer genomics browser analysis

The University of California Santa Cruz (UCSC) Xena browser (http://xena.ucsc.edu/), an analytics, visualization, and Galaxy integration tool for analyzing and viewing public data hubs, was adopted to assess TCGA breast cancer data. For gene expression, RNA-Seq (polyA+ Illumina HiSeq, n = 1218) data was downloaded as log2 (norm_count + 1) values. For the methylation analysis, data from the Illumina Infinium Human Methylation 450 platform was retrieved. This platform expresses DNA methylation as beta values, a continuous variable between 0 and 1 representing the ratio of the intensity of the methylated bead type to the strength of the combined locus [32].

### MEXPRESS tool analysis

The MEXPRESS tool (https://mexpress.be), a user-friendly tool for the visualization and interpretation of TCGA data, provides clinical researchers an easy way to assess TCGA expression, DNA methylation, and clinical data, as well as the relationships among these parameters [33]. The *FOXA1* expression and methylation status in breast cancer were assessed using the MEXPRESS tool.
